# Low-dose antithymocyte globulin plus low-dose posttransplant cyclophosphamide combined with cyclosporine and mycophenolate mofetil for prevention of graft-versus-host disease after HLA-matched unrelated donor peripheral blood stem cell transplantation

**DOI:** 10.1038/s41409-021-01358-y

**Published:** 2021-05-25

**Authors:** Xi Sun, Jun Yang, Yu Cai, Liping Wan, Chongmei Huang, Huiying Qiu, Yin Tong, Xiaowei Xu, Kun Zhou, Xueying Ding, Xianmin Song

**Affiliations:** 1 Department of Hematology, Shanghai General Hospital, Shanghai Jiao Tong University School of Medicine, Shanghai, China; 2 Engineering Technology Research Center of Cell Therapy and Clinical Translation, Shanghai Science and Technology Committee (STCSM), Shanghai, China

**Keywords:** Graft-versus-host disease, Preventive medicine

## Abstract

The standard regimens for graft-versus-host disease (GvHD) prophylaxis in matched unrelated donor (MUD) transplantation were based on antithymocyte globulin (ATG) in combination with calcineurin inhibitors (CNIs). To improve the efficiency of GvHD prophylaxis in MUD peripheral blood stem cell transplantation (MUD-PBSCT), 51 patients with hematological malignancies received a novel regimen for GvHD prophylaxis, which is composed of low dose of ATG (5 mg/kg) plus low-dose posttransplant cyclophosphamide (PTCy, 50 mg/kg) (low-dose ATG/PTCy) combined with cyclosporine A (CsA) and mycophenolate mofetil (MMF). The cumulative incidences (CIs) of grades I–IV and II–IV acute GvHD (aGvHD) were 14.5% (95% CI, 9.4–19.6%) and 6.2% (95% CI, 2.8–9.6%) within 100 days after transplantation, respectively. The CI of mild-to-moderate chronic GvHD (cGvHD) within 1 year was 11.5% (95% CI, 6.6–16.4%). The 1-year probabilities of GvHD and relapse-free survival, relapse-free survival, and over survival were 70.6% (95% CI, 64.2–77.0%), 76.5% (95% CI, 70.6–82.4%), and 82.0% (95% CI, 76.5–87.5%), respectively. The CIs of CMV and EBV reactivation by day 180 were 10.4% (95% CI, 1.5–19.4%) and 8.3% (95% CI, 0.2–16.4%), respectively. The results suggested that low-dose ATG/PTCy combined with CsA/MMF as GvHD prophylaxis in MUD-PBSCT had promising activity.

## Introduction

Graft-versus-host disease (GvHD) remains to be the major complication affecting the survival of patients after allogeneic hematopoietic stem cell transplantation (allo-HSCT) although a substantial progress in reducing acute and chronic GvHD (cGvHD) has been achieved in recent 10 years. Antithymocyte globulin (ATG) in combination with calcineurin inhibitors (CNIs) and either methotrexate (MTX) or mycophenolate mofetil (MMF) has historically represented the standard of care in GvHD prophylaxis for patients undergoing HLA-matched unrelated donor (MUD) transplantation. Nevertheless, the cumulative incidences (CIs) of acute GvHD (aGvHD) were kept in higher levels of 20–50% [1, 2] and cGvHD of 30–50% [3, 4]. So, the best regimens for GvHD prophylaxis need to be explored.

In recent years, posttransplant cyclophosphamide (PTCy) was used for prevention of GvHD in a growing number of clinical trials for patients with HLA-matched related donor (MRD) or MUD transplantation because of its outstanding results of GvHD prophylaxis in haploidentical MRD transplantation. The results of single-agent PTCy as GvHD prophylaxis in HLA-matched bone marrow transplantation (BMT) were unsatisfactory, especially for patients receiving mobilized peripheral blood stem cell (PBSC) grafts [5–7]. PTCy combined with cyclosporine A (CsA) or tacrolimus/MMF for prevention of GvHD in MUD transplantation with PBSC grafts remained to have a relative high incidence of aGvHD with grades II–IV of 28–59% [8–12].

A novel regimen, which consists of a low dose of ATG (5 mg/kg) and low-dose PTCy (one dose of PTCy, 50 mg/kg) (low-dose ATG/PTCy) combined with CNI and MMF, achieved outstanding results for GvHD prophylaxis in haploidentical transplantation [13, 14]. In the current study, we retrospectively analyzed the results of allogeneic PBSC transplantation from MUDs (MUD-PBSCT) to evaluate the efficacy of the novel regimen in GvHD prophylaxis for patients receiving MUD-PBSCT. The results suggested that the novel regimen had excellent outcomes for GvHD prophylaxis in MUD-PBSCT.

## Patients and methods

### Patients, donors, and stem cell sources

The patients undergoing MUD-PBSCT from August 2018 to September 2020 in our center were enrolled into the retrospective study. All patients were diagnosed with hematologic malignancies and lacked of a familiar HLA-related donor. MUD was selected based on HLA-matched grade with no more than two mismatches at HLA-A, -B, and -C, -DRB1, and -DQB1. The PBSC grafts for all patients were mobilized with granulocyte cloning stimulating factor (G-CSF). The target value for CD34^+^ cells in mobilized PBSC graft was a minimum of 4 × 10^6^/kg of recipient weight. MUDs were from China Marrow Donor Program. This study had ethical approval from the local ethical committees and conducted in accordance with the Declaration of Helsinki. All patient data originate from clinical trials with mandatory written informed consent.

### Conditioning regimens and GvHD prophylaxis

The patients above 55 years old (≥ 55 years) received reduced-intensity conditioning (RIC), while the patients below 55 years old (<55 years) received myeloablative conditioning (MAC). The details of conditioning regimens for myeloid malignancies were shown in ref. [13]. All the patients with acute lymphoblastic leukemia (ALL) received MAC regimens, which consisted of busulfan (3.2 mg/kg/day for 4 days), cyclophosphamide (50 mg/kg/day for 2 days), and etoposide (10 mg/kg/day for 2 days) (BCE) or fludarabine (30 mg/m^2^/day) and CTX (25 mg/kg/day for 4 days) (BFC).

The details of low-dose ATG/PTCy combined with CNI and MMF regimen were referred to the ref. [13]. Briefly, the regimen consisted of ATG (2.5 mg/kg/day for 2 days on day −2 to −1) and PTCy (CTX 50 mg/kg/day for 1 day on day +3), CsA and MMF initiating on day +4. CsA was prescribed at 2 mg/kg as a continuous infusion and was tapered from day +60 to day +180. MMF was administered at 15 mg/kg oral three times per day (maximum dose 3 g per day) until day +34 and was then stopped if no aGvHD.

### Engraftment, chimerism monitoring, and GvHD evaluation

Engraftment, complete remissions (CR), and hematologic and molecular relapse were defined by European Society for Blood and Marrow Transplantation (EBMT) criteria [15, 16]. Graft failure was defined according to the literature [17]. Quantitative chimerism monitoring was referred to refs. [18–20]. aGvHD was diagnosed and graded according to the modified Glucksberg grading of aGvHD [21]. cGvHD was diagnosed and graded according to the 2014 National Institutes of Health consensus criteria [22].

### Supportive care

G-CSF was given to all patients since day +5 until neutrophil recovery. All patients received prophylactic levofloxacin and acyclovir from the beginning of conditioning therapy until hematological reconstitution. Prophylactic posaconazole was administered from the day of the conditioning until 1 month after transplant [13, 23]. CMV DNA in serum and EBV DNA in whole blood were routinely monitored and preemptive therapy was performed for CMV and EBV reactivation [13, 24]. EBV related posttransplant lymphoproliferative disease (PTLD) was treated with reducing dose of immunosuppressive agents (IAs) and rituximab [25].

### Statistical analysis

Only patients with successful ANC engraftment were evaluated for aGVHD and cGVHD was evaluated only in patients with a minimum follow-up of 100 days. The CI of relapse was calculated from the date of allo-HSCT or the date of getting CR after transplantation until relapse. Nonrelapse mortality (NRM) was defined as death without evidence of disease relapse. GvHD-free, relapse-free survival (GRFS) events were defined according to the original report as the first event among grades III and IV aGvHD, severe cGvHD, relapse, and death [26]. All statistical tests were two-sided and *P* value < 0.05 was considered significant. The statistical analyses were performed using IBM SPSS 17.0 statistical software (IBM, North Harbour, Portsmouth, UK).

## Results

### Patient characteristics

Fifty-one patients with hematologic malignancies including acute myeloid leukemia (AML), ALL, myelodysplasia (MDS), chronic myelomonocytic leukemia, paroxysmal nocturnal hemoglobinuria, and T-cell lymphoblastic lymphoma underwent MUD-PBSCT from August 2018 to September 2020 in our center. Median age was 36 years (range 19–65 years). At the time of transplantation, 33 patients with AML/ALL reached first or subsequent complete response (CR1, ≥CR2) with conventional therapy or salvage therapy; 12 patients had active disease without responding to salvage therapy. Forty patients received MAC regimens, while eleven patients received RIC regimens. The graft sources were 10/10 MUD for 22 patients (43.1%), 9/10 MUD for 27 patients (53%), and 8/10 MUD for 2 patients (3.9%). In 27 patients with 9/10 MUD, 12 were mismatched at HLA-A locus, 10 at HLA-B locus, 1 at HLA-C locus, 4 at HLA-DRB1 locus, and no patient at HLA-DQ locus. The overall characteristics of the patients and donors are summarized in Table 1.

**Table 1:** Patient demographics.

	Median (range)
Median age	36 (19–65)
Sex	
Male	24
Female	27
Diagnosis	
De novo AML	29
B-ALL	8
MDS-EB II	7
MDS-MLD	1
CMML-II	1
PNH	2
T-LBL	1
T-ALL	2
Disease status at transplantation	
CR1	25
≥CR2	8
Primary refractory	6
Secondary refractory	5
MDS/PNH without treatment	6
T-ALL PR	1
HCT-CI median (range)	0 (0–1)
Conditioning regimens	
MAC	40
RIC	11
HLA match grade	
8/10	2
9/10	27
10/10	22

*AML* acute myeloid leukemia, *MDS-EB II* myelodysplastic syndrome with excess blasts, type II, *CMML* chronic myelomonocytic leukemia, *ALL* acute lymphoblastic leukemia, *T-LBL* T lymphoblastic lymphoma, *T-ALL* T-cell acute lymphoblastic leukemia, *PNH* paroxysmal nocturnal hemoglobinuria, *MDS-MLD* myelodysplastic syndromes-multilineage dysplasia, *CR* complete remission, *MAC* myeloablative conditioning, *RIC* reduced-intensity conditioning, *HCT-CI* Hematopoietic Cell Transplantation-Comorbidity Index.

### Engraftment

All patients received G-CSF mobilized PBSCs with median CD34^+^ cells of 6.23 (2.887–26.1) × 10^6^/kg. Two patients experiencing early death (day +7 and day +10) before engraftment could not be assessed for engraftment analysis, one with septic shock, and another with liver function failure. The median time for neutrophil engraftment was 13 days (range 10–17), while the median time for platelet engraftment was observed in 14 days (range 12–24). The results of chimerism monitoring showed that all of these patients were fully donor chimerism when assessed between days 14 and 28 after transplantation. All evaluable patients successfully engrafted. One patient with AML experienced secondary graft failure after human herpes 6 virus (HHV6) infection with hemophagocytic syndrome at day 30 post transplant.

### Immune reconstitution

**Fig. 1:**
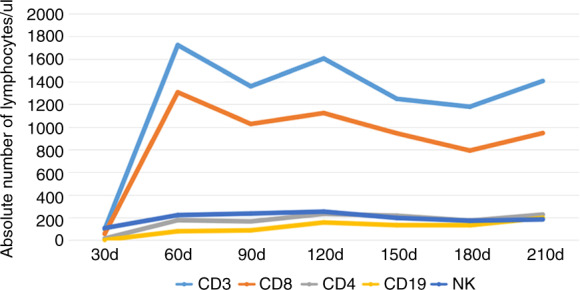
Immune reconstitution after transplantation. Data were shown as median cell counts/μl.

The median lymphocyte counts, stratified by CD3^+^, CD4^+^, CD8^+^, CD19^+^, and CD56/CD16^+^, is depicted in Fig. 1. Forty-one patients were included in immune reconstitution studies and thirty-two cases were analyzed at each endpoint. On days +100 and +120, median CD3^+^, CD4^+^ CD8^+^, CD19^+^, and CD56/CD16+ counts were 1362 (266–2639) and 1607 (831–3591), 169 (75–625) and 236 (109–611), 1030 (328–2346) and 1125 (471–2801), 90 (2–452) and 160 (16–442), 239 (28–1043), and 256 (81–730)/μl, respectively.

### GvHD and other complications

**Fig. 2:**
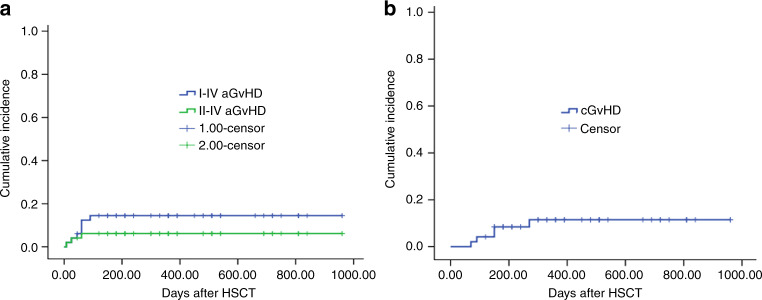
The cumulative incidences (CIs) of graft-versus-host disease (GvHD). **a** The CIs of grades I–IV and II–IV acute GvHD (aGvHD). **b** The 1-year CIs of chronic GvHD (cGvHD).

The CI of grades I–IV aGvHD was 14.5% (95% CI, 9.4–19.6%) and grades II–IV aGvHD was 6.2% (95% CI, 2.8–9.6%) within 100 days after transplantation (Fig. 2a). The frequencies of aGvHD at days +100 and +180 were same due to no patient suffering from late onset aGVHD. Three patients occurred with grade II aGvHD and no patient with grades III and IV aGvHD. Two patients (9/10 matched donors) presented with clinical symptoms of isolated skin and one patient (10/10 matched donor) of skin and gut. All three patients with grade II aGvHD got complete remission after receiving systemic corticosteroids (methylprednisolone with 1.5 mg/kg/d) and anti-CD25 mAbs (Basiliximab, Novartis Pharma AG, Basel, Switzerland) for one patient (10/10 matched donor) because of refractory to corticosteroids. The CI of mild-to-moderate cGvHD within 1 year was 11.5% (95% CI, 6.6–16.4%) (5/48 cases) (Fig. 2b). No patient died from acute and cGvHD.

**Fig. 3:**
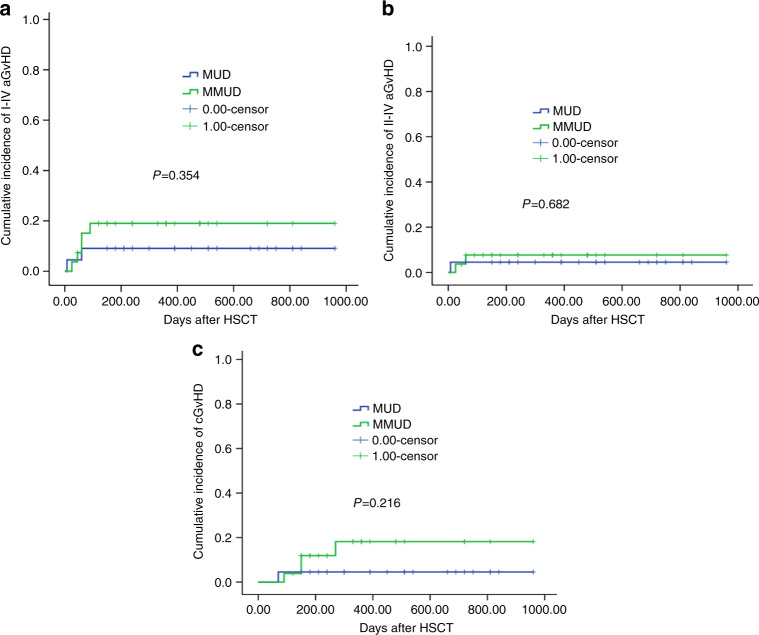
The impact of HLA-matched degree in matched unrelated donor peripheral blood stem cell transplantation (MUD-PBSCT) on graft-versus-host disease (GvHD). **a** The CIs of grades I–IV acute GvHD (aGvHD). **b** The CIs of grades II–IV aGvHD. **c** The CIs of chronic GvHD (cGvHD).

The impact of HLA-matched intensity on GvHD was also analyzed because of 29 patients receiving 1–2/10 loci mismatched MUD (MMUD) transplantation. The CIs of grades I–IV aGvHD were 9.1% (95% CI, 3.0–15.2%) (2/22) in MUD group and 19% (95% CI, 11.4–26.6%) (5/27) in MMUD group, respectively (*P* = 0.354, Fig. 3a), while the CIs of II–IV aGvHD were 4.5% (95% CI, 0.1–8.9%) (1/22) in MUD group and 7.7% (95% CI, 2.5–12.9%) (2/27) in MMUD group (*P* = 0.682; Fig. 3b). The CIs of aGvHD between MUD and MMUD groups were similar. The CIs of cGvHD were 4.5% (95% CI, 0.1–8.9%) (1/22) in MUD group and 18.2% (95% CI, 9.7–26.7%) (4/26) in MMUD group (*P* = 0.216, Fig. 3c).

There were five cases with CMV reactivation and four of five cases soon developed CMV pneumonia without contributing to mortality. Four of these five patients had suffered from grades I and II aGvHD and received systemic corticosteroids treatment. EB virus reactivation was seen in four patients, one of them received CAR-T treatment before transplantation. One patient developed PTLD and recovered from four courses of rituximab. The CIs of CMV and EBV reactivation by day +180 were 10.4% (95% CI, 1.5–19.4%) and 8.3% (95% CI, 0.2–16.4%), respectively. Hemorrhagic cystitis (HC) was diagnosed in four patients with BK virus infection (8.2%) and treated with hydration support symptomatic treatment. Two patients experienced continuous high fever after engraftment and HHV6 sequences were detected in peripheral blood by next-generation sequencing. Both patients recovered after antiviral and high-dose gamma globulin treatment. A total of eight patients (16.3%) suffered from pneumonia (aspergillus pneumonia in one patient, CMV pneumonia in four patient, pneumocystis carinii pneumonia in one patient, tuberculous pneumonia in one patient, unexplained pneumonia in one patient). One patient suffered from hepatosplenic fungal infection 4 months after transplantation and cured with amphotericin B. This patient had a history of lung fungal infection before transplantation (Table 2). No patient developed hepatic sinusoidal obstruction syndrome and transplant associated thrombotic microangiopathy.

**Table 2:** Infectious complications after MUD-PBSCT.

	*N* (%)
CMV viremia	5 (10.4%)
CMV disease	4 (8.2%)
EB infection viremia	4 (8.2%)
PTLD EBV related	1 (2.0%)
Hemorrhagic cystitis BK virus related	4 (8.2%)
HHV6	2 (4.0%)
Pneumonia	
Virus	4 (8.2%)
Fungal	1 (2.0%)
Pneumocystis carinii pneumonia	1 (2.0%)
TB	1 (2.0%)
Unexplained pneumonia	1 (2.0%)
Hepatosplenic fungal infection	1 (2.0%)

*MUD-PBSCT* matched unrelated donor peripheral blood stem cell transplantation, *CMV* cytomegalovirus, *HHV6* human herpes 6 virus, *PTLD* posttransplant lymphoproliferative disease, *TB* tuberculous pneumonia.

### NRM, relapse, and survival

Four patients died of nonrelapse causes. The 1-year probability of NRM was 9.2% (95% CI, 4.1–11.9%) for all patients. Two patients experienced early death (at day +7 and day +10), one with septic shock and another with liver function failure. One patient with AML experienced secondary graft failure at 1 month after HHV6 infection with hemophagocytic syndrome and soon died. One patient died from pneumocystis carinii pneumonia at 3 months after transplantation (Table 3). There was no death due to acute or cGvHD. Five patients died from relapse.

**Table 3:** Causes of death.

Causes of death	No.
Relapse	5
GvHD	0
Infection	2
Secondary graft failure	1
Liver function failure	1

*GVHD* graft-versus-host disease.

**Fig. 4:**
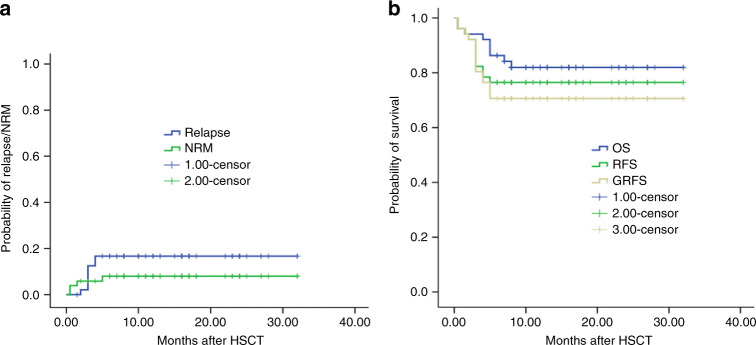
Clinical outcomes after matched unrelated donor peripheral blood stem cell transplantation (MUD-PBSCT). **a** The 1-year probability of relapse and nonrelapse mortality (NRM). **b** The 1-year probability of GvHD-free, relapse-free survival (GRFS), relapse-free survival (RFS), and overall survival (OS).

All 18 patients with active disease achieved CR after transplantation. With a median follow-up of 13 months (range 1.5–32 months), eight cases relapsed. Five of the eight cases were diagnosed with AML or MDS-EB II and all of them were in active status with blasts in bone marrow from 6 to 78% at the time of transplantation. One patient with T-ALL achieved partial remission at the time of transplantation. The median time of relapse was 2 (2–4) months. 3/8 relapsed patients died from relapse without treatments, 1/8 relapsed patient died from relapse after treatment, and 4/8 achieved the second CR (CR2) (Table 4). Within four patients with CR2, one patient reached CR2 after stopping the IAs, one after chemotherapy followed by donor lymphocyte infusion, and another after demethylation drugs combined with interferon therapy. One patient with B-ALL in CR1 developed central nervous system relapse (meningeal invasion) at 3 months after transplantation and achieved CR after intrathecal chemotherapy. The patient getting CR2 through stopping IAs developed moderate cGvHD and died of unexplained lung infection 6 months after transplantation. The 1-year probability of relapse was 16.7% (95% CI, 11.3–22.1%) for all patients (Fig. 4a). The 1-year probabilities of GRFS, relapse-free survival, and overall survival were 70.6% (95% CI, 64.2–77.0%), 76.5% (95% CI, 70.6–82.4%), and 82.0% (95% CI, 76.5–87.5%), respectively (Fig. 4b).

**Table 4:** Status of relapsed patients at the time of transplantation.

	No.
T-ALL PR	1
B-ALL CR1	1
AML with secondary refractory	3
AML with primary refractory	1
MDS-EB II	1
AML CR1 with FLT3-TKD	1

## Discussion

Data from the respective study showed that the CIs of grades I–IV aGvHD and grades II–IV aGvHD within 100 days after transplantation and mild-to-moderate cGvHD within one year were 14.5% (95% CI, 9.4–19.6%), 6.2% (95% CI, 2.8–9.6%), and 11.5% (95% CI, 6.6–16.4%), respectively, in MUD-PBSCT with low-dose ATG/PTCy combined with CsA and MMF regimen for GvHD prophylaxis, which demonstrated the high efficacy of the four drug regimen composed of low-dose ATG, low-dose PTCy, CsA, and MMF in GvHD prophylaxis for patients receiving MUD-PBSCT.

Over recent years, PTCy was added into the GvHD prophylaxis in MUD transplantation to decrease the risk of GvHD because of a high incidence of GvHD with ATG-based regimens [1–4]. The results of single-agent PTCy as GvHD prophylaxis in HLA-matched transplantation were unsatisfactory [5–7]. PTCy with one additional drug of CsA could not effectively prevent the occurrence of aGvHD after MUD transplantation with PBSC grafts. Mielcarek et al. [27] reported the results with PTCy combined with CsA as GvHD prophylaxis for 43 patients with hematological diseases receiving PBSCs from MSD or MUD, although a lower CI of cGvHD and no grades III and IV aGvHD developed, the high incidence of grade II aGvHD over 70% highlighted the importance of optimization of the PTCy-based regimens for GvHD prophylaxis in MUD-PBSCT. The EBMT working group [12] retrospectively analyzed 423 patients with acute leukemia who received PTCy alone (78 cases) and PTCy with one additional immunosuppressive (IS) drug (204 cases) or with two additional IS drugs (141 cases) (−CSA, MTX, or MMF) after MRD or MUD transplantation. The results showed that there were no significant differences in the incidences of aGvHD, 2-year relapse rate, and NRM between the groups. The CI of above grade II aGvHD at 100 days post transplant was 27.9%. Most studies reported that PTCy-based regimens including cyclosporine or tacrolimus/MMF for prevention of GvHD in MUD transplantation with PBSC grafts remained to be with relative high incidences of aGvHD with grades II–IV of 28–59% [8–12]. Our results suggested that low-dose ATG/PTCy combined with CsA and MMF could more effectively prevent aGvHD than PTCy or ATG-based regimens for MUD-PBSCT. The results of low-dose ATG/PTCy combined with CsA and MMF for GvHD prophylaxis in MUD-PBSCT were consistent with previous results in haplo-PBSCT [13, 14].

HLA-matched degree was the most important factor affecting GvHD presence; besides this, there are many other factors such as age, disease status, conditioning intensity, etc. Lorentino et al.’s report [28] showed that with standard PTCy-based regimen for GvHD prophylaxis, the 100-day incidences of grade ≥ 2 and grade ≥ 3 aGvHD were comparable for 10/10 and 9/10 MUD. The 2-year cGvHD and extensive cGvHD were similar between 10/10 and 9/10 MUD. The results suggested that HLA-matched degree did not affect the incidences of GvHD under the platforms of PTCy. In present study, the CIs of aGvHD and cGvHD in patients receiving 10/10 and 8–9/10 MUD transplantation had no significant differences, which suggested that the HLA-matched intensity might not affect the occurrence of GvHD when the novel regimen of low-dose ATG/PTCy combined with CsA and MMF was used for GvHD prophylaxis in MUD-PBSCT. But the results should be carefully interpreted because of the limited number of patients with cGvHD in the study.

All patients excluding two with early death successfully engrafted. The median time for neutrophil engraftment and platelet engraftment were 13 days (range 10–17) and 14 days (range 12–24), respectively. It is well known that mobilized PBSC graft consists of a higher content of mononuclear cells, CD34^+^ cells, and T cells compared with BM graft, which may lead to faster hematopoietic and immune reconstitution, however the using of PTCy appeared to negate the reconstitution advantage of PBSC grafts. Moiseev et al. [11] reported that in MUD-PBSCT with PTCy-based GvHD prophylaxis regimens, the median implantation time of neutrophils and platelets was significantly longer than that with ATG-based regimen. In our previous studies, low-dose ATG/PTCy combined with CNI and MMF regimen did not affect the hematopoietic reconstitution time when compared with ATG-based regimen in haplo-PBSCT [13, 14]. In our present study, we found that low-dose ATG/PTCy combined with CsA and MMF regimen also did not affect the hematopoietic recovery speed in MUD-PBSCT. We speculated that the addition of low dose of ATG (5 mg/kg) at pre transplantation would ensure the engraftment and fasten the hematopoietic reconstitution.

Mortality from GvHD and infection accounted for the vast majority of NRM in allo-HSCT. In our study, NRM was only 9.2%, which was relatively lower when compared with PTCy regimen (16%) or ATG-based GvHD prophylaxis regimens (36%) [11]. As per follow-up till now, only two patients had developed moderate cGvHD with only skin involvement and no patient died from GvHD. Only two patients died from infection. These results showed that low-dose ATG/PTCy combined with CsA and MMF regimen could reduce NRM through effectively preventing GvHD. In our study, median CD4^+^ lymphocyte counts were 169/μl on day +100, which were higher than that with ATG-based regimens (100/μl) at the same time, but on day +120, CD4^+^ lymphocyte counts with 236/μl reached the level on day +180 days (200/μl) in ATG-based group [29]. These data indicated that immune recovery might be faster in our study than with ATG-based regimens in MUD transplantation. Although four cases developed CMV pneumonia, none contributed to mortality. EB virus reactivation was only seen in four patients and one patient developed PTLD. HC associated with BK virus infection presented in 8.2% (4/49) of patients, which was lower than that in patients receiving PTCy-based or ATG-based regimens for GvHD prophylaxis [11]. Our data in corporation with these results at least suggested that low-dose ATG/PTCy had a relative high safety profile.

Disease recurrence after all-HSCT is still a major concern when GvHD was successfully controlled, especially for patients who are not remission at the time of transplantation. In present study, only eight cases relapsed and five of them were in active disease at transplant. The median relapse time was 2 (2–4) months after transplantation, which indicated that the early relapse was related with the preconditioning effects, but not with graft-versus-leukemia (GVL) effects. Low-dose ATG for GvHD prophylaxis in MRD transplantation did not increase the relapse risk, which has been demonstrated in several clinical trials [30–32]. PTCy has been demonstrated in preclinical experiments that it could separate GvHD and a GVL effect [33] and did not increase the relapse risk in clinical studies [34, 35]. Our group previously compared the results of low-dose ATG/PTCy with standard ATG-based regimens in the haplo setting and found that low-dose ATG/PTCy combined with CNI and MMF did not increase the relapse risk. These results suggested that low-dose ATG/PTCy combined with CsA and MMF might not increase the relapse risk of patients after MUD transplantation, but remains to be further explored with longer follow-up and more samples.

In conclusion, this is a pilot study to establish a low-dose ATG with low-dose PTCy combined with CsA and MMF as an effective regimen to prevent GvHD in MUD-PBSCT with a higher survival. But this study still had several limitations, including a lower number of samples, a shorter follow-up, disease heterogeneity, etc. Thus, further studies with high methodological quality, such as larger sample sizes and randomized and controlled trials, are required to compare the efficacies of this regimen with ATG or PTCy-based regiments in MUD-PBSCT.

### Ethics and dissemination

Results from this investigation will be presented in peer-reviewed international journals. In addition, major findings will be disseminated at national and international conferences. In addition, we will bring out major findings to the general public.
